# Pitfalls in the conceptualization of primary palliative care and the WHO public palliative care framework

**DOI:** 10.1017/S1478951525100229

**Published:** 2025-06-16

**Authors:** Juan Esteban Correa-Morales, Rebecca Newell, Libby Sallnow

**Affiliations:** 1 Marie Curie Palliative Care Research Department, University College London, London, UK; 2 Public Health Department, Brighton and Sussex Medical School, Brighton, UK

Despite decades of advocacy for palliative care as a public health priority, millions of people worldwide still lack access to it and continue to suffer without adequate support at the end of life. Progress has been patchy, inconsistent, and primarily concentrated in higher-income countries (World Health Organization 2024a). In this context, the World Health Organization (WHO) promotes two key principles: expanding primary palliative care (PPC) and measuring the effectiveness of palliative care delivery (Rajan et al. 2024; World Health Organization 2018). However, defining PPC – as well as the models and indicators used to develop and measure its effectiveness – remains contested and shaped by unique historical, social, and systemic challenges. Ultimately, how we define PPC will determine what we choose to measure and which interventions are prioritized going forward.

PPC has been defined as care provided by primary healthcare workers, who serve as the main point of support for people with serious illnesses within their communities. This approach emphasizes early palliative interventions – ideally delivered at home or close to home – in collaboration with specialized palliative care services when available, alongside the continued development of primary care providers’ palliative care capabilities (Munday et al. 2019). Given the diversity of primary healthcare systems globally – particularly in regions such as Asia, Africa, and South America – and the proven success of community-led interventions in other health areas like maternal care and HIV/AIDS, it is surprising that current definitions of PPC do not adequately recognize the vital role of community-centered approaches (Peeler et al. 2024). Community-based interventions have enabled effective, sustainable, and community-sensitive approaches to HIV prevention and management. These have empowered communities, improved health, and helped reduce stigma and barriers in discussing sensitive topics (Salam et al. 2014).

The omission of community-based interventions from PPC reflects a broader conceptual challenge: when PPC is understood primarily as a clinical intervention led by healthcare professionals, indicators tend to focus on quantifiable clinical metrics, such as the number of providers trained or the volume of morphine administered, while overlooking informal, community-driven aspects that are often central to care in many parts of the world.

These healthcare-centered indicators fail to capture crucial dimensions such as caregiver burden, community engagement, and death literacy – factors that are essential to palliative care provision and to communities facing life-limiting conditions. Despite providing an estimated 95% of home-based care, caregivers are frequently excluded from dominant health metrics (Sallnow et al. 2022). Yet their well-being, shaped by geography, socioeconomic conditions, housing, and community support, is deeply intertwined with the quality and sustainability of palliative care and must be recognized as a core component of holistic, person- and community-centered PPC (Sallnow et al. 2022). PPC should encompass social and civil community initiatives, recognizing that the majority of care for those facing serious illness, death, and dying takes place in the community through a combination of formal and informal networks. Although this broader perspective is gradually gaining traction, the absence of established indicators to measure the impact of community-based initiatives remains a significant barrier to their meaningful inclusion in policy, practice, and advocacy aimed at strengthening palliative care.

The WHO’s model of public health for palliative care development does not propose any indicators related to death literacy in the education dimension. In the community empowerment dimension, the model includes two indicators: the first assesses whether advocacy groups exist to promote the rights of patients, families, caregivers, and disease survivors, though it includes specialized medical associations among these groups; the second evaluates the presence of guidelines or policies for advance care planning (ACP), although ACP is not necessarily a focus of community-based initiatives and its overall effectiveness remains highly debated (World Health Organization 2021). A significant demographic divide also exists in who completes ACP, with lower rates among ethnic minority groups, persons with low income, and those with limited health literacy. This raises important questions about the equity and global relevance of ACP as a universal strategy (Ashana et al. 2022; Morrison et al. 2021).

There remains a critical gap in how we understand the broader social and emotional impacts of community-led initiatives on people facing serious illness, death, and grief (Abel and Kellehear 2022). If we continue to frame PPC only through clinical lenses, we risk deepening inequalities and overlooking the very networks that sustain care where health systems often cannot. Death is not only a medical event; it is a deeply social experience. To truly meet the needs of diverse communities, health systems, practitioners, and researchers must reimagine PPC as both a clinical and civic responsibility. This means developing indicators that reflect lived experiences, investing in community capacity, and embedding social support as a core element of care. Only then can we build a more inclusive, equitable, and holistic palliative care future.
